# Effectiveness of upgraded maternity waiting homes and local leader training in improving institutional births among women in the Jimma zone, Ethiopia: study protocol for a cluster-randomized controlled trial

**DOI:** 10.1186/s13063-019-3755-z

**Published:** 2019-12-04

**Authors:** Jaameeta Kurji, Manisha A. Kulkarni, Lakew Abebe Gebretsadik, Muluemebet Abera Wordofa, Sudhakar Morankar, Kunuz Haji Bedru, Gebeyehu Bulcha, Kednapa Thavorn, Ronald Labonte, Monica Taljaard

**Affiliations:** 1 School of Epidemiology and Public Health, University of Ottawa, 600 Peter Morand Crescent, Ottawa, ON K1G 5Z3 Canada; 2 Department of Health, Behaviour & Society, Jimma University, Jimma Town, Jimma Zone Ethiopia; 3 Department of Population & Family Health, Jimma University, Jimma Town, Jimma Zone Ethiopia; 4 Jimma Zone Health Office, Jimma Town, Jimma Zone Ethiopia; 5 Ontario Hospital Research Institute, The Ottawa Hospital - General Campus, Ottawa, ON Canada; 6 Ontario Hospital Research Institute, Ottawa Hospital, Civic Campus, 1053 Carling Ave, Civic Box 693, Admin Services Building, ASB 2-004, Ottawa, ON K1Y 4E9 Canada

**Keywords:** Ethiopia, Maternal health care, Maternity waiting home, Institutional birth, Cluster-randomized controlled trial, Complex interventions, Community engagement, Three-Delays model, RE-AIM framework

## Abstract

**Background:**

Ethiopia is one of the ten countries in the world that together account for almost 60% of all maternal deaths. Recent reductions in maternal mortality have been seen, yet just 26% of women who gave birth in Ethiopia in 2016 reported doing so at a health facility. Maternity waiting homes (MWHs) have been introduced to overcome geographical and financial barriers to institutional births but there is no conclusive evidence as to their effectiveness. We aim to evaluate the effects of upgraded MWHs and local leader training in increasing institutional births in the Jimma zone of Ethiopia.

**Methods:**

A parallel, three-arm, stratified, cluster-randomized controlled trial design is being employed to evaluate intervention effects on institutional births, which is the primary outcome. Trial arms are: (1) upgraded MWH + religious/community leader training; (2) leader training alone; and (3) standard care. Twenty-four primary health care unit catchment areas (clusters) have been randomized and 3840 women of reproductive age who had a pregnancy outcome (livebirth, stillbirth or abortion) are being randomly recruited for each survey round. Outcome assessments will be made using repeat cross-sectional surveys at baseline and 24 months postintervention. An intention to treat approach will be used and the primary outcome analysed using generalized linear mixed models with a random effect for cluster and time. A cost-effectiveness analysis will also be conducted from a societal perspective.

**Discussion:**

This is one of the first trials to evaluate the effectiveness of upgraded MWHs and will provide much needed evidence to policy makers about aspects of functionality and the community engagement required as they scale-up this programme in Ethiopia.

**Trial registration:**

ClinicalTrial.gov, NCT03299491. Retrospectively registered on 3 October 2017.

## Background

The recently established Sustainable Development Goals reaffirm a global commitment to reducing maternal mortality [1]. Significant progress was made in reducing maternal mortality worldwide during the Millennium Development Goal period from 1990 to 2015; however, levels remain unacceptably high and large regional disparities exist. Globally, maternal mortality in 2015 was 216 per 100,000 livebirths, while in sub-Saharan Africa the rate was more than double this (546 per 100,000 livebirths). Ethiopia is one of the ten countries in the world that together account for almost 60% of all maternal deaths [2].

The majority of maternal deaths are preventable if women have timely access to good-quality maternal health-care services. Access to skilled obstetric care during and soon after birth is critical for the survival of women. However, in Ethiopia, only 26% of women who gave birth in 2016 reported doing so at a health facility [3]. In fact, over 40% of births were overseen by traditional birth attendants and in 15% women were entirely on their own [3]. Obstetric services are provided at the health centre and hospital level but are not available at community-based health posts.

Several barriers have been identified in rural sub-Saharan African that can impact a woman’s ability to access skilled obstetric care. These include barriers in the decision to seek care, barriers in reaching care, and barriers in receiving quality care once they arrive at a health facility, commonly referred to as the “three delays” [4]. Community-based interventions are often implemented to address the first two barriers [5–7], while the third barrier requires health system improvement on several levels [8].

Geographical and financial barriers are frequently cited as barriers to reaching skilled obstetric care during and after birth [9]. Women regularly have to travel large distances across difficult terrain, often made impassable during the rainy seasons, to get to health facilities [10]. Transportation options are frequently limited and may be expensive, leading to a limiting effect on the utilization of obstetric care [11]. Community-based surveys in Ethiopia report that women who live closer to health facilities are more likely to give birth there; for instance, women who are within a 1-h walking distance have 3.3 times higher odds of delivering at a health facility [12].

To address physical accessibility issues, particularly in rural areas where health facilities equipped with emergency obstetric services are sparsely distributed, maternity waiting homes (MWHs) have been constructed near or within health facilities. Women approaching their delivery date who will have difficulty in reaching a health facility on time are temporarily accommodated in these “homes away from home”. Women who are at a high risk for complications during delivery, such as very young mothers, women expecting twins, and those diagnosed with conditions such as high blood pressure, are also often referred to MWHs [13].

In Ethiopia, the Federal Ministry of Health has developed explicit guidelines pertaining to MWHs that outline referral criteria, minimum standards for accommodation and services to be provided, strategies to mobilize community contributions, and roles of various levels of government in managing MWHs. The challenge, however, lies with implementation and providing an acceptable level of service quality. A national facility assessment in 2012 on all MWHs listed in Federal Ministry of Health records found that the majority of MWHs did not provide food and did not have attendants to clean and maintain the MWHs [14]. Lack of space to accommodate relatives who were relied upon for food supplies and the absence of staff at night and during weekends were among the complaints made. The quality of MWH facilities has also been shown to affect institutional delivery rates in other African countries [15]. Women also expect health workers to check on them while they are at the MWHs and to assist with transfer to delivery rooms when they go into labour. With the effort and expense it takes to come to the MWHs, an absence of antenatal care support at an MWH could make staying at home a preferable option [16].

Community and religious leaders have been reported to be invaluable in mobilizing communities in order to improve access to health services in Ethiopia [17]. Additionally, given the prominent role of community contextual factors such as social norms around institutional births, community beliefs and expectations, and autonomy of decision-making by women, engaging local leaders in efforts to improve the access to care for women is crucial [9, 18].

This cluster-randomized trial is designed to evaluate the effects of upgraded MWHs and local leader training combined, or local leader training alone, versus usual care on the number of institutional births. We hypothesize that both interventions will increase the proportion of women who have institutional births, with the combined intervention expected to result in a greater increase. As secondary objectives, we will evaluate the effect of the interventions on antenatal care and postnatal care utilization and compare the costs and health outcomes associated with each intervention from the societal perspective.

## Methods

### Setting

The trial is being conducted in three rural districts (Gomma, Seka Chekorsa and Kersa) in the Jimma zone located in southwestern Ethiopia. These districts were selected from among the 18 districts located in the Jimma zone because: 1) they had the largest available populations; 2) MWHs were present at health centres; and 3) they did not have any active maternal and child health interventions at the time.

### Intervention components

Two interventions are being evaluated in the trial: upgraded MWHs and local leader training combined, and local leader training alone.

#### Upgraded MWHs

Existing MWHs will be provided with supplies to create a home-like environment suitable for pregnant women to reside in prior to delivery. The supplies provided were selected based on a review of existing literature, a rapid-needs assessment conducted at baseline, and input from the Jimma Zone Health Office (JZHO). Eleven indicators are used to specify the minimum required services at MWHs and these include room(s) with the capacity for at least 10 women, bedding, a kitchen with a chimney, a food supply, materials for the coffee ceremony (an important cultural routine for families), a clean water supply, a power source, toilet facilities, a bathing area, an attendant for the MWH, and follow-up by a skilled birth attendant. Items supplied are listed in Table 1.

**Table 1:** Supplies provided to upgrade maternity waiting homes (MWHs) as part of the MWH intervention component

Bedding	Utensils	Personal hygiene	Other
Mattresses	Coffee grinder	Bath towels	Solar lamps
Bed sheets	Glasses	Buckets	Water tank (1000 l)
Pillows	Plates	Slippers	Cooking stove
Blankets	Water jug	Soap	Broom
	Coffee cups	Washing powder	Plastic floor sheets
	Coffee pot	Sanitary pads	Mop
	Local bread pan		Bleach
	Pots		Drinking water purifier

A register was also codesigned with the JZHO and introduced to facilitate tracking of MWH users and services provided as outlined in the national MWH policy guideline. The register is managed by antenatal care nurses who refer women to MWHs and are responsible for monitoring pregnant users during their stay prior to delivery. Funds are also provided to employ an attendant to clean the MWH, prepare meals and assist users during their stay at the MWH.

#### Local leader training

Community-based health extension workers (HEWs), religious leaders, and community leaders (specifically the Women’s Development Army) are targeted for the training workshops. The training aims to facilitate identification of barriers to accessing maternal health-care services and strategies to overcome these. The workshops use participatory learning methods that build on individual experiences. HEWs, who are employed by the Ethiopian health system, are invited to attend 3-day workshops while religious and local leaders attend 1-day workshops. Content for the workshops were developed by the research team based on barriers to care identified in the Three Delay Model and through formative, qualitative research carried out in 2016 [19]. Leaders are expected to integrate what they learn into their routine engagement with the communities to promote safe motherhood practices including accessing maternal health-care services and use of MWHs.

### Standard care (control group)

MWHs were officially introduced around 2013 and have gradually been scaled up across the country. These are modelled as a community–government partnership with a significant reliance on community contributions for their sustainability. Communities make both cash and in-kind contributions in the form of coffee and/or food. There is large variation in the availability of supplies and quality of services provided amongst MWHs. This is partly due to a fluctuation in resources available during the year among families and partly due to a weak management system. No records are maintained of MWH users and, in practice, there is very little monitoring of women who stay at these facilities. JZHO data suggest that, at baseline, the majority of MWHs in the study area were either very poorly or poorly functional based on the 11 service indicators. The national guidelines specify that women who live at a distance from health facilities or cannot be reached by ambulance or are 38 weeks pregnant or more and at risk of experiencing obstetric complications should be referred for MWH stay [20]. However, women are typically referred to MWHs when they present with false labour or arrive at the very early stages of labour. A smaller proportion of women are referred to MWHs by antenatal care nurses if they live very far away from a health centre and are expected to deliver in a few weeks. Referral practices vary among health centres and staff.

Health promotion activities are mainly conducted by HEWs who are sometimes assisted by members of the Women’s Development Army. Although some religious leaders may encourage their community members to deliver at health facilities, this is not a widespread or systematic practice in the study districts.

### Trial design

**Fig. 1:**
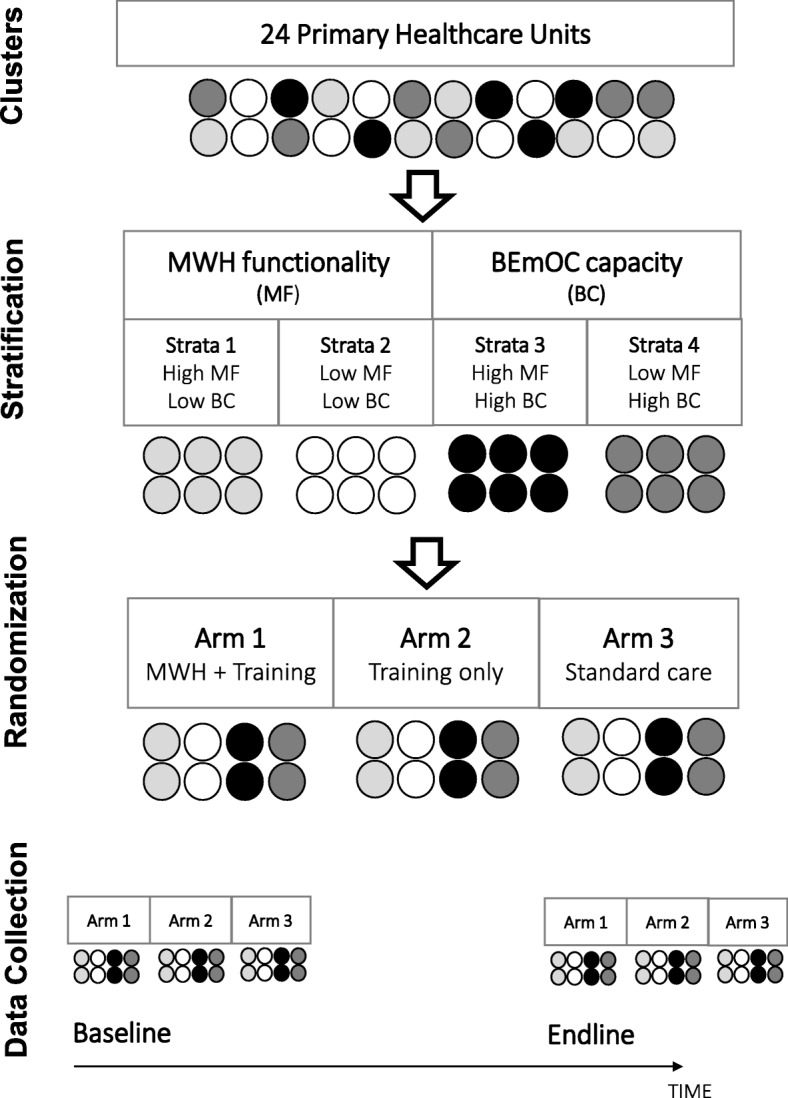
Schematic of trial design. BEmOC basic emergency obstetric care, MWH maternity waiting home

This study is a parallel, three-arm, stratified, cluster-randomized controlled, superiority trial with 24 clusters. The trial arms are as follows: 1) upgraded MWH + leader training; 2) leader training alone; and 3) standard care. Primary health-care unit (PHCU) catchment areas were designated as clusters for the trial. PHCUs are composed of a health centre and satellite health posts; health posts operate in the community, covering a population of 3000–5000 and are each managed by two to three HEWs. MWHs are located within health centres as standalone structures or in the form of a room assigned to function as an MWH. Outcome assessments will be made using repeat cross-sectional surveys at baseline (prior to intervention roll-out) and at 24 months postintervention (i.e. the endline). A schematic for the trial design is displayed in Fig. 1. A cluster-randomized design was selected because the interventions are delivered at the health facility and community level which precludes individual-level randomization.

### Cluster and individual selection

PHCUs were eligible for trial participation if health centres had a standalone MWH or a room designated for this purpose. All 26 health units in the three districts were eligible and 24 were randomly selected for the trial using a random number generator in STATA v13.

Women of reproductive age were eligible to participate in the trial if they were living in the villages within the selected PHCU catchment areas and had a pregnancy outcome (livebirth, stillbirth, spontaneous/induced abortion) up to 12 months prior to a survey round; baseline surveys commenced in October 2016 and endline surveys are scheduled to begin in April 2019. Lists of pregnant women registered by HEWs at health posts and Women’s Development Army volunteers within villages (‘*kebeles*’) function as the sampling frame for selection of eligible women at each survey time point. Names of women, their village of residence and their date of delivery organized by PHCU are included in the sampling frame. Random numbers generated in STATA v13 were assigned to each woman in the list, ranked, and then the required number sequentially selected. Since women were not excluded based on prior participation in surveys, it is possible that there is some overlap of participation in baseline and endline surveys, but the probability is expected to be low.

### Intervention assignment and masking

Stratification was employed to ensure a balanced distribution of poorly functioning MWHs and low basic emergency obstetric care (BEmOC) capacity health centres between the trial arms. JZHO data on MWH functionality assessed using the 11 indicators were used to classify clusters as high functioning (≥5 of 11 MWH indicators present) or low functioning (<5 of 11 MWH indicators present). Clusters were also grouped based on their capacity to provide BEmOC; high-capacity clusters were those that had at least 5 of the 7 signal functions present while low-capacity clusters had less than 5 signal functions present according to 2016 JZHO data. Signal functions are essential obstetric interventions, such as provision of parenteral anticonvulsants, necessary to prevent maternal deaths; they are used to assess the level of obstetric care provided at health facilities [21].

Clusters were stratified into four groups based on these strata (low MWH + low BEmOC; low MWH + high BEmOC; high MWH + low BEmOC; high MWH + high BEmOC). Within each stratum, a random number generator in STATA was used to generate the allocation schedule. The allocation sequence was generated by MAK who was not involved in implementing the trial and it was shared in a password-protected document with the principal investigator in Ethiopia (LAG) who was also not involved in recruitment and enrolment of clusters or individuals. Random allocation of clusters to trial arms took place once all clusters had been recruited for the study.

Interviewers collecting outcome data are blind to intervention assignment. Due to the nature of the intervention it is not possible to blind women or health-care providers at PHCUs to their intervention status. However, all women and health-care providers are blind to the study hypotheses. The consent for data collection includes a general description of the overall aims of the study (namely to understand the experiences of women when they are pregnant, giving birth and after delivery and to look for ways to make this safer for women and their babies), but women are not aware of their cluster’s allocation to the intervention or control arms.

### Participant timeline

**Fig. 2:**
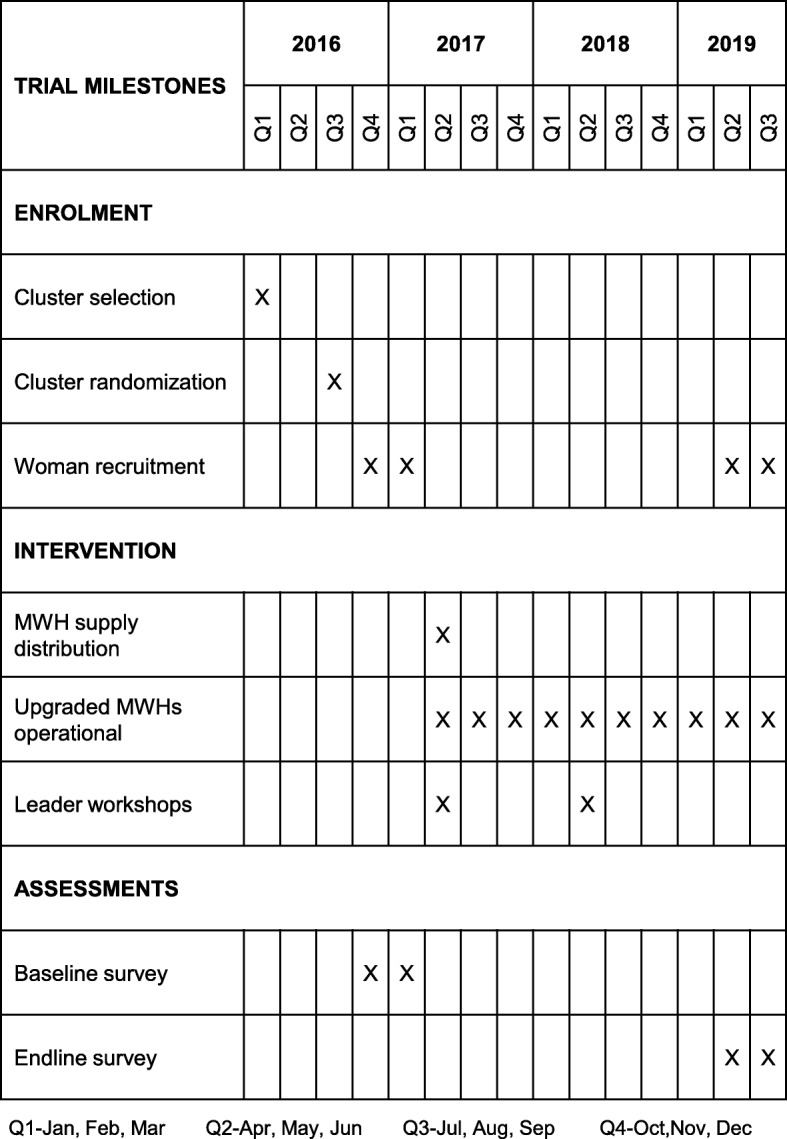
Tentative trial timeline. MWH maternity waiting home

Clusters were enrolled in March 2016 and randomized to trial arms in September 2016. Baseline recruitment and interviewing of women within study clusters began in October 2016 and was completed by January 2017. Distribution of materials to the MWHs and training of leaders and HEWs commenced in May 2017; upgraded MWHs were operational in June 2017. The endline assessments are scheduled to start in April 2019. An outline of the trial timeline is illustrated in Fig. 2.

In terms of participation in outcome assessments, women who are randomly selected for interviews at baseline and endline typically spend 25 min on average giving consent and about an hour for interviews.

### Sample size

Methods outlined by Hooper and Bourke [22] for parallel-arm, cluster-randomized trials with repeated cross-sections were used to calculate the sample size (see Additional file 1). Briefly, the methodology requires calculation of two design effects, with the product of the two used to inflate the sample size under individual randomization to account for within-period intracluster correlation coefficient (ICC) and the between-period ICC. The within-period ICC is the correlation between any two women in the same cluster and the same period, while the between-period ICC is the correlation between any two women in the same cluster but different periods.

The first design effect (d_c_) due to cluster randomization was calculated using a within-period ICC of 0.1 obtained from a review of community-based, cluster-randomized controlled trials in low-resource settings focusing on maternal health-care service outcomes [23]. The design effect was calculated as:

$$ {\mathrm{d}}_{\mathrm{c}}=1+\left(\mathrm{m}-1\right)\uprho $$

where m is the cluster-period size (i.e. the number of women surveyed per PHCU in each round) and ρ is the within-period ICC.

The second design effect (d_r_) due to repeated assessments (baseline and endline) was calculated using both the within-period ICC and a cluster autocorrelation coefficient (π) of 0.8 to allow for a 20% decay of the correlation from within to between different periods [22]. We had no prior information to inform the cluster autocorrelation coefficient, but a 20% decay was considered reasonable. The second design effect was calculated as:

$$ {\mathrm{d}}_{\mathrm{r}}=\left(1-{\mathrm{r}}^2\right) $$

where r = (mρπ)/d_c_.

The sample size assuming individual randomization was then multiplied by both design effects to arrive at a required sample size of eight PHCUs per arm with an average of 160 women per PHCU per round of survey, for a total sample size of 3840 for each survey (total women recruited = 7680). This sample size achieves 80% power to detect an absolute difference in the proportions of institutional births of 0.17 assuming a control arm proportion of 0.4 and using a two-sided alpha of 0.025 to account for two pairwise comparisons. The control arm proportion was obtained from JZHO data. An absolute difference of 0.17 is the smallest difference that can be detected, i.e. the difference between the weakest intervention (hypothesized to be the leader training intervention) arm versus control.

### Outcomes

The primary outcome is self-reported institutional birth defined as delivery of the last child at a health facility where obstetric care is provided (i.e. health centre or hospital) as reported by an enrolled woman. Births at home, en route to a facility or at a health post will not be considered an institutional birth.

Two other maternal health-care service outcomes will be assessed: 1) antenatal care and 2) postnatal care. Self-reported antenatal care received for last child delivered as well as the total number of antenatal care visits made will be assessed. Self-reported postnatal care received for the last child delivered within 48 h and 6 weeks will be assessed as secondary outcomes.

### Data collection and management

Data on outcomes and other variables of interest are collected through household surveys prior to intervention roll-out (baseline) and after 24 months of implementing the intervention (endline). Trained interviewers will conduct face-to-face interviews using structured questionnaires programmed onto tablet computers using Open Data Kit. Questionnaires contain sections on sociodemographics, reproductive history, maternal health-care service use, danger sign knowledge, attitudes towards maternal health-care services, awareness of/experience with MWHs, social support and health-related quality of life. Interviews are expected to last approximately 1 h and will be conducted in a quiet, private space at the homes of the women. If selected women are absent from their homes, interviewers will visit households up to three different days/times to attempt to interview them before the woman will be replaced with another randomly selected woman. Some demographic information will be collected from those women who refuse to take part in the trial if they agree to provide this data.

Data collected are submitted to a secure cloud server on a weekly basis from tablet computers. They are checked for inconsistencies and errors and these are communicated to field supervisors and brought to the attention of interviewers to avoid repetition and preserve data quality.

Qualitative data will be collected at baseline and at follow-up. Focus group discussions (with religious leaders and local community leaders) and key informant interviews (with HEWs and health facility staff) will be conducted to understand the roles stakeholders play in maternal and child health, what activities they engage in to promote safe motherhood and what their expectations and experiences are with respect to MWHs. As part of process monitoring, routine meetings held at the district and community level will be periodically attended over the course of the 24-month intervention period; data will be collected primarily using participant observation and field notes.

### Primary analysis: effectiveness of the intervention components

Baseline characteristics of women will be tabulated by trial arm to provide an overview of the study population and to check for any notable imbalances. Characteristics of interest will include the age of women, the education levels of women, the distance between home and the nearest health centre, and household wealth. Means, standard deviations and ranges will be presented for continuous variables, while frequencies and percentages will be reported for categorical variables.

An intention to treat approach will be employed where random assignments of clusters to the three trial arms will be preserved regardless of adherence to the intervention assignment. The primary outcome will be analysed using a generalized linear mixed model with random effects for PHCU (cluster) and time, and fixed effects for time, time by trial arm interaction, and the stratification variables. The main effects for trial arm will be dropped to constrain differences between the arms at baseline as recommended by Hooper et al. [24]. With only two measurement points, it will not be possible to determine whether or not time has a more complex relationship with the outcome (quadratic or cubic) and therefore only a first-order term for time will be included. To account for the bias due to a small number of clusters, we will use the Kenward–Roger degrees of freedom approximation [25].

A logit link function will be used, and the outcome assumed to have a binomial distribution. Pairwise comparisons of adjusted least square mean differences will be made together with 97.5% confidence intervals to determine the effect of each intervention arm versus control.

Odds ratios will be calculated by taking the exponential of the relevant combinations of regression coefficients. The ICC and cluster autocorrelation coefficient for institutional births (primary outcome), antenatal care use, and postnatal care use will be calculated in STATA and reported on the proportions scale. Secondary outcomes will be analysed as described for the primary outcome.

Statistical tests and confidence intervals will be two-sided; between-group comparisons will be calculated and presented with 95% confidence intervals with the significance levels set at the 2.5% level.

### Secondary analysis: understanding implementation for scale-up

Due to the pragmatic nature of the trial and an interest in supporting scale-up, if found to be effective, the RE-AIM framework is being used to guide secondary analyses focusing on implementation. Briefly, the impact of an intervention is assessed by evaluating reach, efficacy, adoption, implementation, and maintenance [26]. Reach and efficacy measures are captured in our primary and secondary trial outcomes; adoption, implementation and maintenance of the intervention components will be gauged using qualitative data collected from HEWs, health centre staff, and district and zonal health office staff. Uptake of the intervention components, changes in policy implementation and financial support as well as mechanisms used to sustain the interventions that develop during the trial will be examined. Finally, the short- and long-term cost-effectiveness of the intervention components will be determined using a decision analytic modelling approach. The short-term cost-effectiveness analysis will be based on trial data as well as data specific to the Jimma zone and Ethiopia. This short-term study will provide an incremental cost per intermediate outcomes, including the number of women who have institutional births. A Markov model will be developed and used to simulate the natural history of pregnancy, pregnancy-related complications, and the long-term cost-effectiveness of maternal and neonatal interventions of interest over the lifetime period of women. The outcomes of the long-term model will be expressed as an incremental cost per an additional life year saved. We will conduct extensive sensitivity analyses, including both deterministic and probabilistic sensitivity analysis with Monte-Carlo simulations, to assess the robustness of our model to parameter uncertainty. Where possible, we will utilize the regression analyses on baseline sociodemographic characteristics associated with cost and outcome and stratify the simulated cohort to reflect these sources of variability in cost-effectiveness outcomes.

## Ethics and dissemination

### Research ethics approval

Ethical approval was obtained from the University of Ottawa Health Sciences and Science Research Ethics Board (File no. H10–15-25B) and the Jimma University College of Health Sciences Institutional Review Board (Ref. no. RPGE/449/2016). Informed consent will be obtained from all study participants.

### Protocol amendments

The trial was initially designed with three rounds of survey, but due to budget constraints the protocol was changed to include only two surveys (baseline and endline). This was done after the baseline survey but prior to analysis. This resulted in the minimum absolute detectable difference increasing from 15% to 17% to maintain other sample size parameters (80% power, ICC = 0.1 and 24 clusters with 160 women each). An economic analysis component was also included (protocol version 3.0, January 2016) and consent forms simplified as per ethics board feedback (protocol version 2.0, October 2015).

### Informed consent

In this setting, there is a close relationship between the community and administrative structures at various levels. The JZHO primarily formulates health policies and works closely with the district health office which in turn supervises implementation and service delivery at the PHCU level. The community is actively engaged through community-based HEWs and the Women’s Development Army which regularly interfaces with the PHCU staff. Through this cascade, information about the trial was informally shared by Jimma University partners (who independently have a respected, long-standing relationship with the community through their research and development work) and approval for cluster participation secured prior to commencing the study. Concerns about which clusters would be allocated to intervention at the district level were addressed by explaining the importance of preserving random allocation and by assurances that interventions found to be effective would be scaled-up.

Verbal informed consent for data collection is obtained from eligible women willing to participate in interviews prior to either round of household surveys. Trained interviewers read out the contents of the consent forms outlining the survey objectives, institutions and investigators involved and describing what is expected of women as well as associated risks and benefits. This is done in a local language of choice (Amharic or Afaan Oromo). Women are also explained their rights as participants and their questions answered prior to enrolment. Since clusters are randomized before the surveys, women who consent to take part in surveys will be providing consent after randomization has already taken place. It is not possible to obtain individual consent for study interventions as the interventions are delivered at the level of the entire community.

### Confidentiality

The names of the women, the village of residence and point location of dwelling are collected on encrypted questionnaires and stored separately from the rest of the collected data. Names are collected to detect and correct errors in study identification number assignment. Point locations are collected for planned spatial analyses distinct from intervention effectiveness analysis, the latter being the primary focus of this trial. Only the principal investigators in Jimma, Ethiopia, and Ottawa, Canada, have access to this personal identifier information. Data shared with the research team for analysis purposes will be de-identified first by removing the names of the women.

### Dissemination plan

A National Advisory Committee consisting of individuals from institutions such as the Federal Ministry of Health, Ethiopian Public Health Institute and the Ministry of Science and Technology is being engaged to enhance policymaker participation and to promote future uptake of effective interventions in a sustainable manner. Annual meetings are held to brief the committee on progress and a final dissemination meeting will be held towards the last few months of the trial. Scientific papers highlighting various study results will also be published in open-access journals.

## Discussion

Our study will be among the first of the few trials to assess the effectiveness of MWHs in improving institutional births [27]. There have been several observational studies conducted to evaluate various aspects of MWH effectiveness. Lower maternal mortality and stillbirths among MWH users was reported in a retrospective cohort study in Ethiopia [28]; a hospital-based cohort in Zimbabwe described a higher relative risk of perinatal death among women who delivered at home compared to those who were admitted to a health facility through MWHs [29]; a matched-cohort study in Liberia found an increase in the proportion of skilled deliveries in facilities with MWHs compared to controls [30]. However, these designs had inherent biases that arose mainly because assignment to exposure was driven by specific factors that may contribute to observed outcomes making causal inference difficult; group comparability was also uncertain. These studies suggest that MWHs have the potential to improve both the coverage of institutional births as well as maternal health outcomes. However, there is a need to generate more reliable evidence using stronger designs such as randomized controlled trials [27].

In terms of design, the risk of contamination has been minimized by randomizing at the PHCU level where both intervention components are mainly delivered. Randomizing at the cluster level, however, can introduce selection bias when recruiters have knowledge of cluster allocation [31]. To minimize this risk, we are using HEW community lists of pregnant women to randomly select individuals within recruited clusters for surveys. Significant time and resources have been invested to ensure these lists are up to date and complete. Despite the logistical challenges of deploying interviewer teams to scattered locations resulting from random selection of women, selections were maintained to preserve trial integrity.

To ensure balance between trial arms and group comparability, clusters were stratified by both MWH functionality and the BEmOC capacity of health centres. BEmOC capacity was used as a proxy for quality of care, which has been reported to affect the primary outcome of institutional births [32–34]. Tablets on which survey data were collected were programmed with required questions to minimize missing outcome data. Interviewers are not able to proceed with the survey if they do not enter responses. Missing data and cluster withdrawal are often of concern in cluster-randomized trials [35]; however, we do not expect cluster withdrawal because policy makers and programme implementers who function as both important stakeholders and community gatekeepers are partnering in the trial. It is possible that women may switch clusters by seeking services outside of their catchment area; however, this is a pragmatic trial aiming to measure effectiveness to inform practice and therefore needs to be able to accommodate such eventualities. We anticipate some nonresponse (due to individuals who decline to participate in the survey) although this is anticipated to occur at random and nondifferentially across the arms. Nevertheless, we will assess the extent of selection bias that may be present by comparing the demographic profile of respondents and nonrespondents.

By combining upgraded MWHs and leader training intervention components, our trial has limited ability to detect the effect of upgraded MWHs alone on increases in institutional births. However, given the influential effects of the community and context on the willingness and ability of women to access maternal health-care services [36–39], a pragmatic approach that integrates an intervention component to create an enabling environment for women is likely more appropriate. Many women require permission from their husbands to stay at MWHs while others need someone to step in to handle domestic responsibilities and take care of children to enable them to stay at an MWH. Absence of support in these areas hinders some women from using MWHs [14, 16, 40, 41] as it does, more generally, in accessing other maternal health-care services. A lack of awareness of the existence of MWHs and their benefits can also contribute to low usage [16]. Negative perceptions associated with MWH use among communities can discourage women from staying at MWHs [42]. Local religious and community leaders can function as change agents who, if engaged, can positively influence beliefs and practices and mobilize support for women [43, 44].

While the overall goal of any efforts to improve the access of women to maternal health-care services is to reduce maternal mortality, our trial does not have the resources to support the sample size that would be required to detect a change in this relatively rare outcome. We therefore selected institutional births as our primary outcome; this focus aligned well with maternal health indicators used by the Ethiopian Federal Ministry of Health and was endorsed as a useful metric to guide policy implementation and appropriate resource allocation. We also relied on self-reporting by women of institutional births, which may be subject to some limitations due to poor recall.

Finally, embedding the intervention components within the health system with an explicit link to the community should help to facilitate scale-up. The results of this trial will provide much needed evidence to policy makers about the effectiveness and cost-effectiveness of functional MWH and local leader training in improving utilization of maternal health-care services with the overarching goal to reduce maternal mortality.

## Trial status

Participant recruitment began on 15 October 2016 and baseline data collection was completed in January 2017. Intervention implementation began in June 2017 and is currently on-going. The endline survey is scheduled to begin in April 2019 and is anticipated to be completed by July 2019, at which time recruitment will also be complete. This is protocol version 3.0, dated 12 January 2016.

## Supplementary information

**Additional file 1.** Sample size calculation.

## Data Availability

Not applicable.

## Abbreviations

BEmOC: Basic emergency obstetric care
HEW: Health extension worker
ICC: Intracluster correlation coefficient
JZHO: Jimma Zone Health Office
MWH: Maternity waiting home
PHCU: Primary health-care unit
