# Supplementary material for: Effectiveness of upgraded maternity waiting homes and local leader training in improving institutional births among women in the Jimma zone, Ethiopia: study protocol for a cluster-randomized controlled trial
Source: Trials. 2019 Dec 4;20:671. doi: 10.1186/s13063-019-3755-z (PMC6894194; doi:10.1186/s13063-019-3755-z)
Supplement: Supplementary file 1 — Additional file 1. Sample size calculation. [file 13063_2019_3755_MOESM1_ESM.docx]

**SUPPLEMENTARY INFORMATION**

**Sample Size Calculations**

Assumptions:

- The proportion of institutional births expected in control areas was assumed to be 40% based on data from the Jimma Zone Health Office. The target difference was an absolute increase of 0.17.
- The power to detect an absolute difference was fixed at 80% while the family-wise alpha was set at 0.025 to account for two pairwise comparisons.
- The available number of clusters was also fixed as only 26 PHCUs were present in the three study districts. We therefore varied cluster size per period (m) to evaluate what would be feasible for the trial.
- The sample size for an individually-randomized trial with a simple parallel design was calculated in STATA using a family-wise alpha of 0.025 and was calculated to be 163 women per arm.
- We chose a within-period ICC of ρ=0.1 which is just above the midpoint of the range of published estimates for our primary outcome from other settings (0.025-0.15).

In the table below, we vary the within-period (ρ) and the cluster autocorrelation coefficient (π) to demonstrate how the required sample size changes.

| **Cluster size per period (m)** | **Cluster autocorrelation coefficient (π)** | **Within-period ICC (ρ)** | **Number of clusters per arm** | **Total number of participants per arm**  **(n)** |
| --- | --- | --- | --- | --- |
| 150 | 0.7 | 0.025 | 5 | 1,500 |
| 160 | 0.7 | 0.025 | 4 | 1,280 |
| 200 | 0.7 | 0.025 | 4 | 1,600 |
| 150 | 0.7 | 0.1 | 11 | 3,300 |
| 160 | 0.7 | 0.1 | 11 | 3,520 |
| 200 | 0.7 | 0.1 | 10 | 4,000 |
| 150 | 0.7 | 0.15 | 15 | 4,500 |
| 160 | 0.7 | 0.15 | 15 | 4,800 |
| 200 | 0.7 | 0.15 | 14 | 5,600 |
| 150 | 0.8 | 0.025 | 4 | 1,200 |
| 160 | 0.8 | 0.025 | 4 | 1,280 |
| 200 | 0.8 | 0.025 | 4 | 1,600 |
| 150 | 0.8 | 0.1 | 8 | 2,400 |
| 160 | 0.8 | 0.1 | 8 | 2,560 |
| 200 | 0.8 | 0.1 | 8 | 3,200 |
| 150 | 0.8 | 0.15 | 11 | 3,300 |
| 160 | 0.8 | 0.15 | 11 | 3,520 |
| 200 | 0.8 | 0.15 | 11 | 4,400 |
| 150 | 0.9 | 0.025 | 4 | 1,200 |
| 160 | 0.9 | 0.025 | 4 | 960 |
| 200 | 0.9 | 0.025 | 4 | 1,200 |
| 150 | 0.9 | 0.1 | 6 | 1,800 |
| 160 | 0.9 | 0.1 | 6 | 1,920 |
| 200 | 0.9 | 0.1 | 5 | 2,000 |
| 150 | 0.9 | 0.15 | 7 | 2,100 |
| 160 | 0.9 | 0.15 | 7 | 2,240 |
| 200 | 0.9 | 0.15 | 7 | 2,800 |
